# Supplementary figures and images for: A pipeline for assembling low copy nuclear markers from plant genome skimming data for phylogenetic use
Source: PeerJ. 2022 Dec 6;10:e14525. doi: 10.7717/peerj.14525 (PMC9745922; doi:10.7717/peerj.14525)

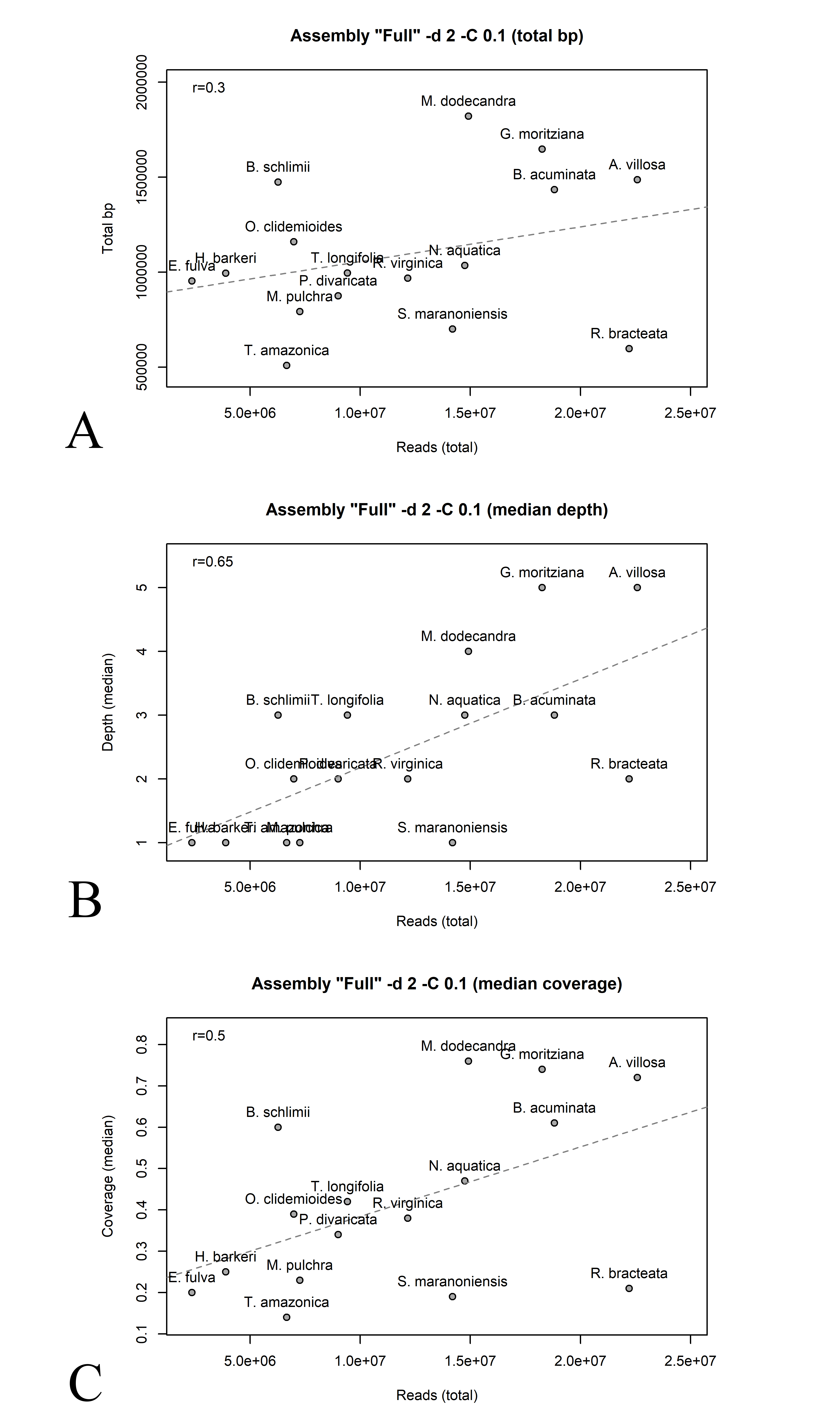

Supplement: Supplemental Information 1 [file peerj-10-14525-s001.png]

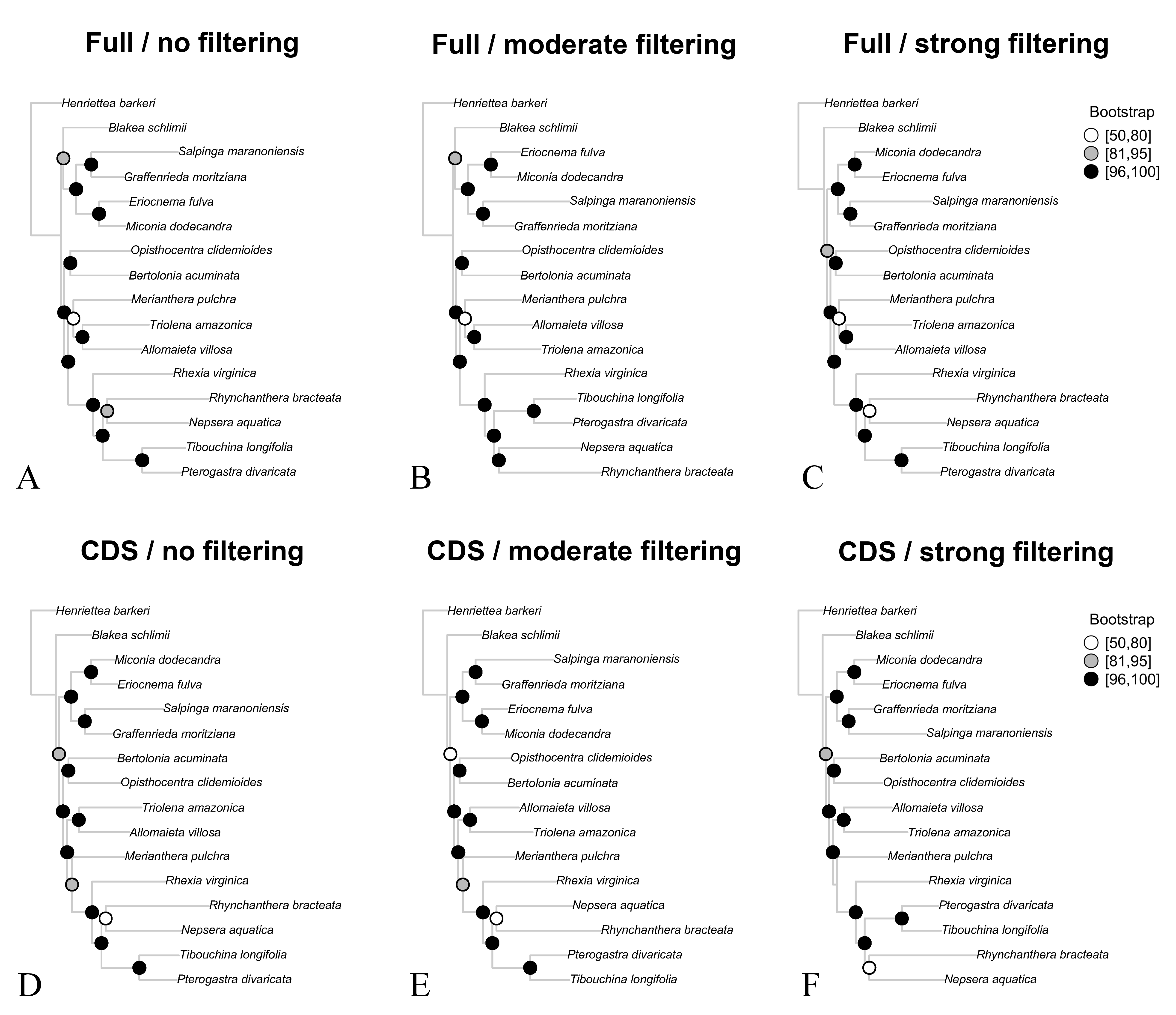

Supplement: Supplemental Information 2 — A. References = full, filtering = none. B. References = full, filtering = moderate. C. References = full, filtering = strong. D. References = transcripts, filtering = none. E. References = transcripts, filtering = moderate. F. References = transcripts, filtering = strong. [file peerj-10-14525-s002.png]

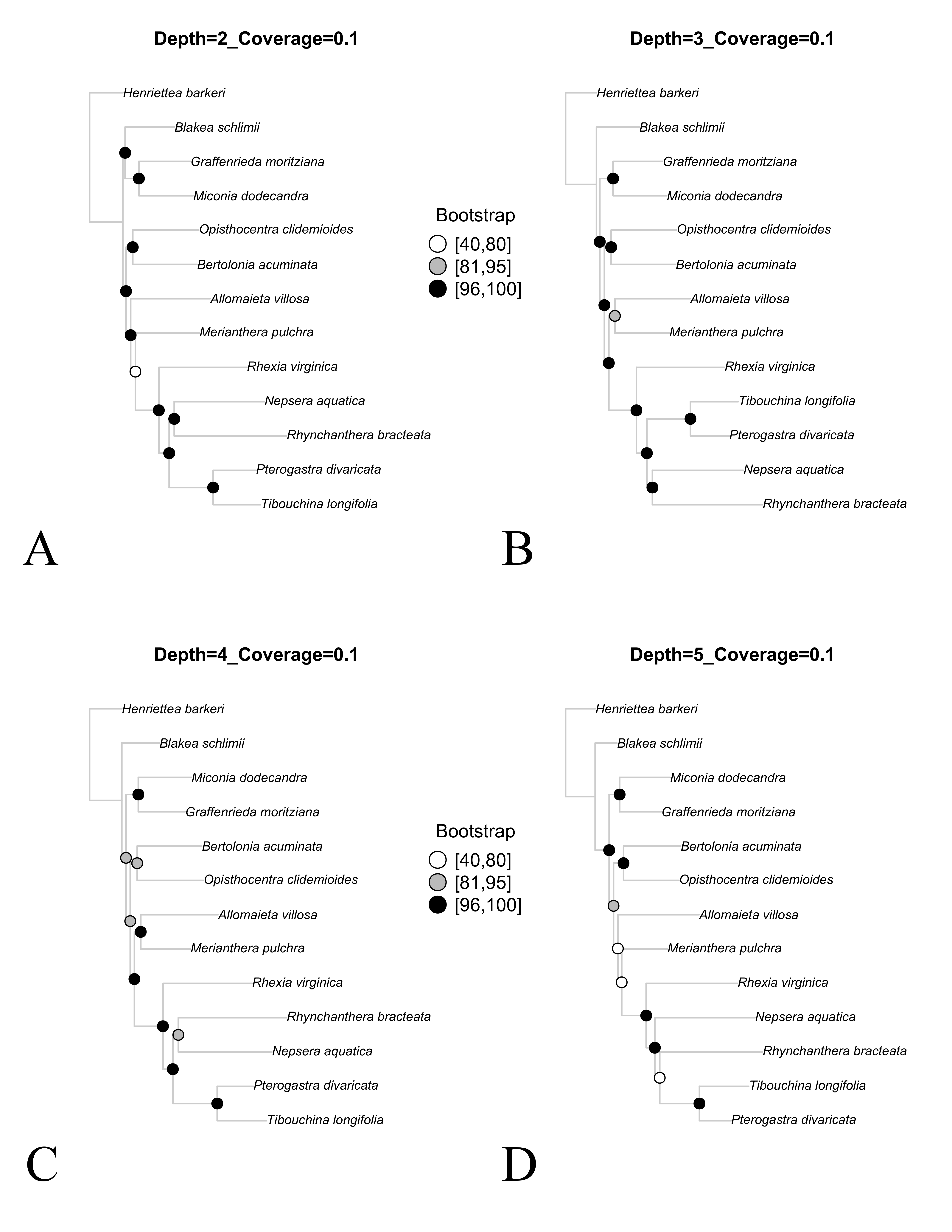

Supplement: Supplemental Information 3 — A. Depth = 2. B. Depth = 3. C. Depth = 4. D. Depth = 5. [file peerj-10-14525-s003.png]

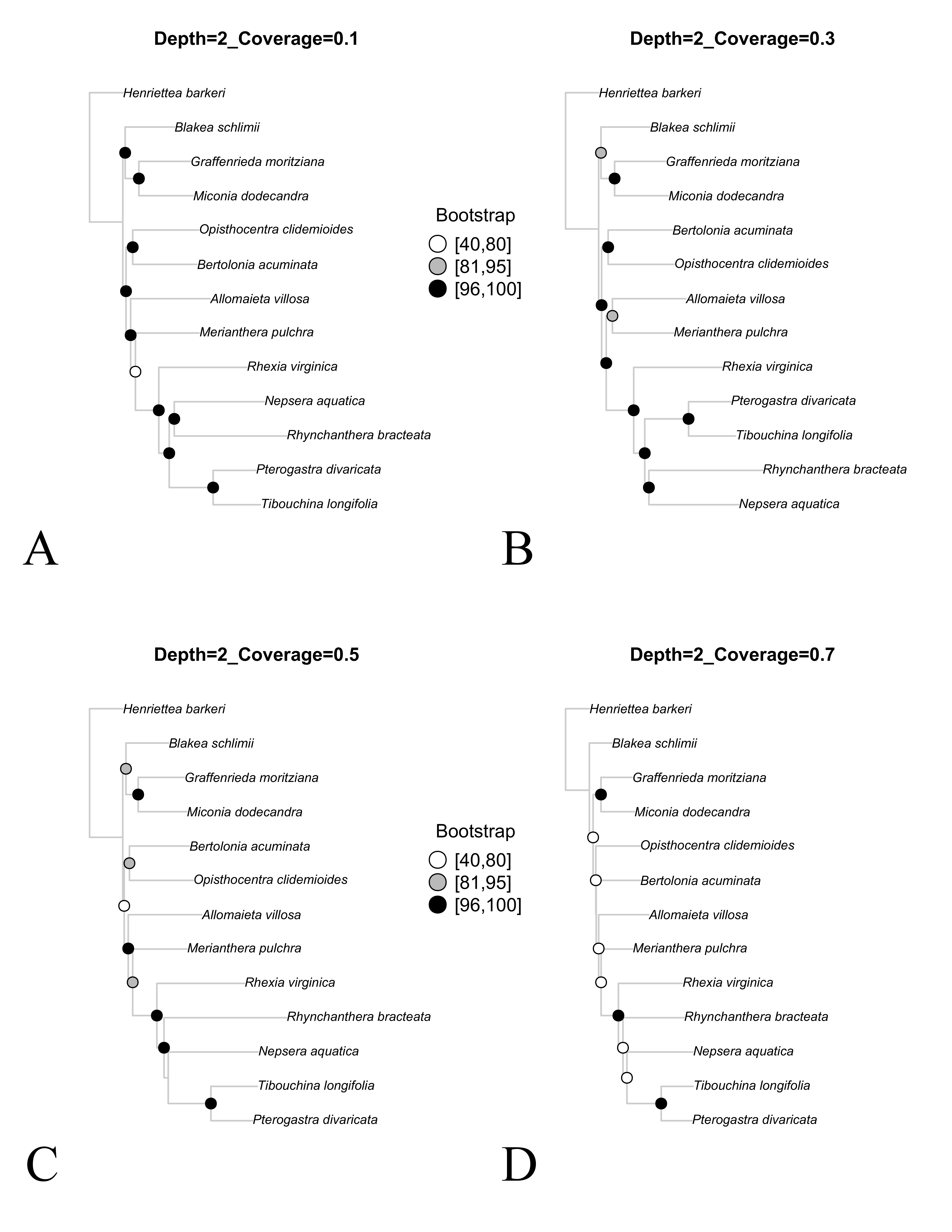

Supplement: Supplemental Information 4 — A. Coverage = 0.1. B. Coverage = 0.3. C. Coverage = 0.5. D. Coverage = 0.7. [file peerj-10-14525-s004.png]

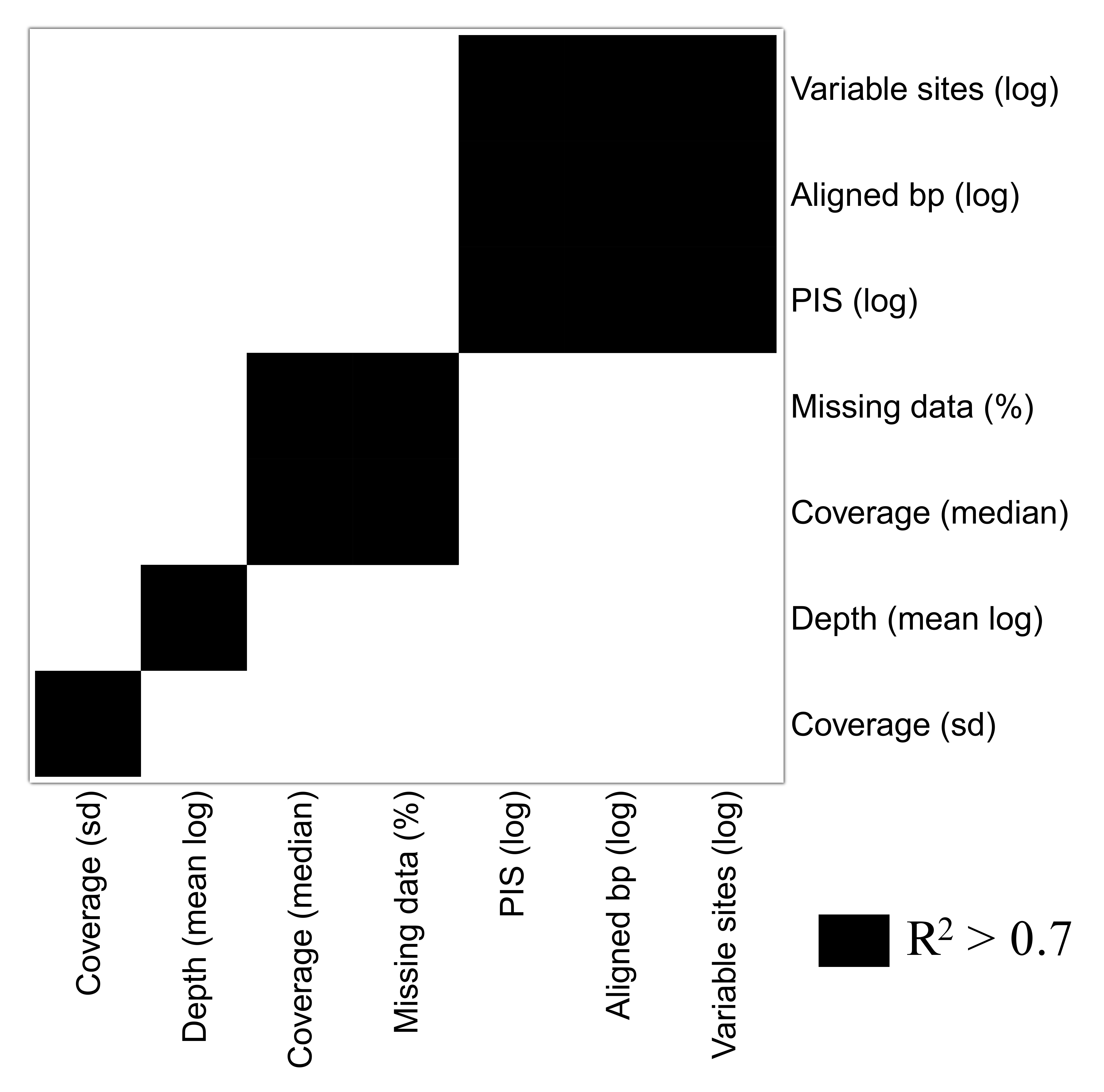

Supplement: Supplemental Information 5 [file peerj-10-14525-s005.png]

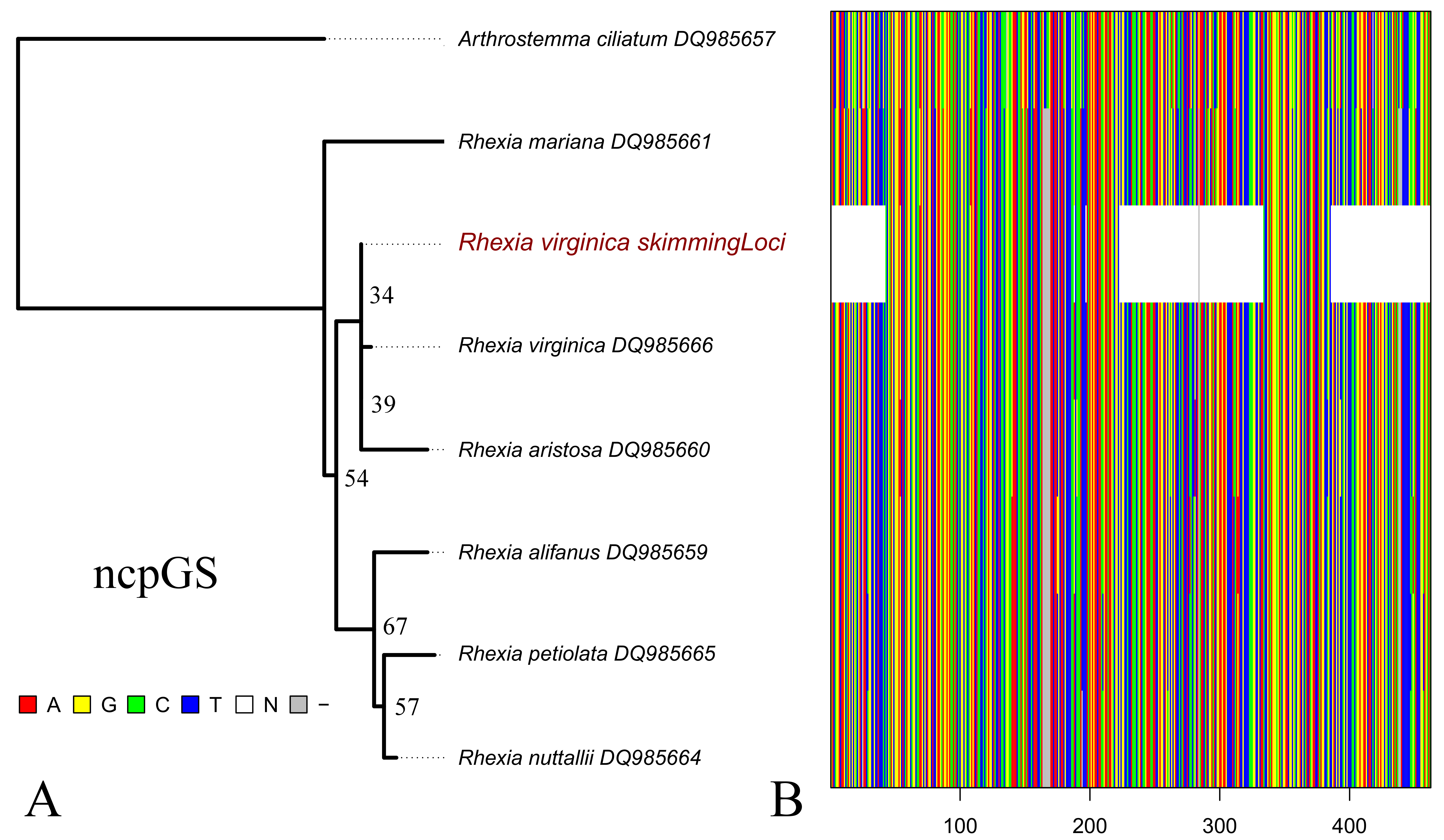

Supplement: Supplemental Information 6 [file peerj-10-14525-s006.png]
